# Pandemic-Related Challenges and Organizational Support Among Personnel in Canada's Defense Establishment

**DOI:** 10.3389/fpubh.2021.789912

**Published:** 2022-01-27

**Authors:** Irina Goldenberg, William James Denomme, Jennifer E. C. Lee

**Affiliations:** Department of National Defence, Director General Military Personnel Research and Analysis, Ottawa, ON, Canada

**Keywords:** military, COVID-19, pandemic, organization, work-life balance

## Abstract

In the final week of March 2020, 2.8 million Canadians were away from their usual places of work and engaging in remote and/or telework to mitigate the spread of COVID-19 (Statistics Canada, 2020). The Government of Canada's Department of National Defence (DND) and the Canadian Armed Forces (CAF) were no exception, with most members from the regular force (Reg F), the primary reserve force (P Res), and the DND public service (DND PS) working from home. The COVID-19 Defence Team Survey was administered from April 29th, 2020, and May 22nd, 2020, to gain insight into work, health, and family-related challenges since the onset of the pandemic and change in work arrangements. Responses from five open-ended questions were qualitatively analyzed to determine general themes of concern regarding work, personal, and family related challenges, stress-management and coping strategies, and recommendations for improving the work situation and personal well-being. Given the different roles and conditions of employment, responses of the different groups or “components” of respondents (Reg F, P Res, DND PS) were compared to identify common and unique challenges to inform targeted organizational responses. A total of 26,207 members (Reg F = 13,668, 52.2%; P Res = 5,052, 19.3%; DND PS = 7,487, 28.6%) responded to the survey's five open-ended questions, which yielded a total of 75,000 open-ended responses. When asked about work-related challenges, respondents' most common challenges included dissatisfaction with technology/software, work arrangements, ergonomics, work-life balance, communication within the organization, and the uncertainties regarding career development. In terms of personal and/or family-related challenges, the most common challenges included social isolation, the impact of the pandemic on mental health, school closures and homeschooling, caring for vulnerable family members, and childcare concerns. The most common stress-management and coping strategies included exercise, spending time outdoors, communicating or spending time with family members, household chores/projects, mind-body wellness exercises, and playing games. The most common recommendations made by respondents to improve their work- or personal-related situations included improving technological capabilities, streamlining communication, providing hardware and software necessary to ensure comfortable ergonomics, the provision of flexibility in terms of telework schedules, return-to-work decisions, and the expansion of benefits and access to childcare services. In terms of differences among the components, DND PS personnel were most likely to report dissatisfaction with technological changes and ergonomics, and to recommend improving these technological limitations to maximize productivity. Reg F members, on the other hand, were most likely to recommend increased support and access to childcare, and both Reg F and P Res members were more likely to mention that increased benefits and entitlements in response to the COVID-19 pandemic would be ameliorative. The results of this study highlight several important facts about the impact of the COVID-19 pandemic on personnel working in large, diverse organizations. For example, advancements in organizational technological capabilities were highlighted herein, and these are likely to grow to maintain productivity should remote work come to be used more extensively in the long-term. This study also highlighted the importance of flexibility and accommodation in relation to individual needs – a trend that was already underway but has taken on greater relevance and urgency in light of the pandemic. This is clearly essential to the organization's role in supporting the well-being of personnel and their families. Clear and streamlined communication regarding organizational changes and support services is also essential to minimize uncertainty and to provide useful supports for coping with this and other stressful situations.

## Introduction

The COVID-19 pandemic has been a devastating event with revolutionary implications for Canadians and the world. At the time of writing of this manuscript, over 4.75 million individuals worldwide have died due to complications caused by this virus (1), of which 27,695 deaths occurred in Canada alone (1). In addition, the majority of Canadians were subjected to restrictive measures to reduce the spread of the virus, including stay-at-home orders and the closure of schools, daycares, and non-essential businesses (2). While these measures were necessary, they have also resulted in prolonged periods of social isolation; an economic crisis comparable to that of the 2008 recession (3–6); heightened rates of unemployment across a broad range of sectors (6, 7); and an unprecedented proportion of Canadians working from home [~32% of Canadians, compared to ~4% before the onset of the pandemic; (7–10)].

Like most Canadian organizations, military and national defense establishments were affected by the COVID-19 pandemic. The majority of civilian personnel within the Department of National Defence (DND) and military personnel in the Canadian Armed Forces (CAF) were required to suddenly and quickly work from their homes through telework, remote work, and alternative work arrangements at unprecedented levels (11–15). DND and CAF personnel were likely to be similarly affected by the various challenges stemming from the pandemic, such as concerns over health and safety, social isolation, reduction in the availability of important services, and concerns over the well-being of children and other loved ones. As such, the DND/CAF developed empirical research to help understand the challenges and experiences of their personnel in order to inform organizational approaches to address these issues and support its personnel. This article presents the findings of this research, focusing on DND/CAF civilian and military personnel's challenges with, and adjustment to, the COVID-19 pandemic, particularly in relation to their work and personal well-being.

To date, many studies have assessed how civilian workers have adjusted to working from home, particularly from a productivity standpoint. Fortunately, this research has consistently found that workers have “adjusted well” to work-related changes. Nine in 10 workers working from home in Canada reported being at least as productive as when they worked in their usual location (9), mirroring those working from home in the United States (16, 17) and the United Kingdom (18, 19). Workers have also reported greater autonomy, reduced occupational stress, and increased motivation for their work while working from home (20, 21). Furthermore, individuals working from home were spared some of the concerns of those who were not able to work from home (i.e., frontline and essential workers), including a heightened fear of COVID-19 transmission (22, 23), dissatisfaction with safety precautions in the workplace (23), and lack of access to personal protective equipment (24, 25). Despite these benefits, surveys of Canadians working from home found that equal proportions wanted to return to pre-pandemic work arrangements, continue to work from home permanently, or adopt a hybrid approach (9, 17, 19).

This variability suggests that there have been various challenges to employees' productivity and well-being at work that must be acknowledged and understood. Researchers have warned that working from home, telework, and alternative work arrangements can have adverse consequences if they are mandatory (26). These potential challenges include organization unpreparedness, communication challenges and job sharing limitations, and the unsuitability of working from home for certain workers (26, 27). Such challenges can foster toxic relationships and dissatisfaction in the remote workspace (26–28). Indeed, since the onset of the pandemic, Canadians working from home reported that isolation from co-workers, limited access to work-related resources/information, unsuitable ergonomic arrangements, and technological limitations (e.g., software/hardware unavailability, slow internet speed) acted as barriers to their work-related well-being and productivity (9, 24, 28). These challenges were mirrored in other countries as well, along with technological limitations (29), communication challenges with co-workers (29–31) and managers/senior leaders (29), and unsuitable ergonomics (32–34). Employees also reported disruptions in work-life balance (29, 35–38). These disruptions could be due to a greater inability to disconnect from one's work, a lack of distinction between one's work environment and home environment, working longer hours, and higher workloads (9, 28, 29, 39), and personal insecurity about one's productivity and performance (29, 30, 40).

The personal and family health of personnel has also suffered because of the pandemic. Many individuals, both in and outside of Canada, have reported decreases in their mental health, feelings of loneliness, anxiety, depression, panic, and overall psychological distress (41–48). There has also been an increased prevalence of burnout in those working from home (35, 49). Furthermore, studies have highlighted reduced opportunities and options for physical exercise, resulting in increased sedentary behavior (50–54) and limited options to cope and maintain one's physical health amid the pandemic (55). Given that most families have been confined to their homes and isolated from friends and extended family, there may also be negative impacts on familial well-being, including increased family conflict, dissatisfaction, and even violence (56–60), particularly when there is financial stress on the household (61) or for those facing substance use issues (57). Finally, homeschooling and constant childcare have also been incredibly demanding, leading to further infringement of work-life balance and leaving little room for leisure and entertainment (62, 63).

A minimal amount of research has examined adjustments of military personnel to the COVID-19 pandemic (64), and existing research has focused mainly on mental health outcomes (64). Therefore, the current study examines a broad range of challenges to both work and personal well-being among CAF military personnel, and compares these to civilian personnel working from the DND using the COVID-19 Defence Team Survey, administered in the spring of 2020. This survey was designed to “better understand Defence Team members' experiences and needs related to the COVID-19 pandemic, with a view to identify organizational approaches for supporting personnel and their families today and in the future” [(15); p. 1]. Over 27,000 members of the DND/CAF responded to this survey of more than 60 close-ended questions, highlighting the myriad challenges during the COVID-19 pandemic (15). While previous work has presented the findings from the close-ended survey responses, respondents of the COVID-19 Defence Team Survey also provided input on several core issues in their own words through their answers to five open-ended questions. Given that the scale of the pandemic and the response to it are unprecedented in recent times, these open-ended questions were created to tap into potentially unrecognized and unpredictable elements that may not have been covered by the close-ended survey questions. In addition to assessing challenges and concerns, these open-ended questions probed coping approaches and what the organization could do to support its employees. Here, we report on the findings from the analysis of employees' responses to these open-ended questions to complement the insights uncovered using close-ended questions.

Research has also shown that the effects of the pandemic can vary as a function of individual characteristics, including gender, age, ethnicity, income, and family status (22, 23, 65), and may depend on the type of work and employment sector individuals engage in (22, 28). As a result, one could surmise that personnel from different groups in DND/CAF may differ in terms of the dynamic work and personal consequences of the pandemic. Three broad subgroups or *components* of personnel comprising the DND/CAF Defence Team are regular force (Reg F) military personnel, Primary Reserve Force (P Res) military personnel, and DND civilian Public Service (DND PS) personnel. Reg F military personnel serve the CAF in a full-time manner as a profession. Primary reservists are military members who are generally employed by the armed forces to complement or supplement regular force military capacity, often on a part-time basis, while also being employed in the civilian labor market (66, 67). DND PS personnel perform a wide variety of tasks and are found in many different occupations within defense establishments, including administrative support, technical, scientific, and professional positions (68). Civilians are also often employed within senior leadership and executive roles, sharing the responsibility for the management and leadership of defense establishments with their military counterparts (69, 70). In light of the systemic differences between components, the current study also compared the primary concerns of personnel from these respective components. Ultimately, the insights observed in this study are intended to help inform organizations—both military and civilian—to identify and understand challenges to their personnel's well-being and productivity, explore how these might vary across different segments of the workforce, and shed light onto approaches that might be used to address these challenges throughout the ongoing pandemic and in response to potential future crises.

## Method

In the context of a comprehensive survey battery comprising closed-ended items, the COVID-19 Defence Team Survey also included five open-ended questions asking respondents to share their perceptions and experiences in their own words (15, 71). The survey was accessible to all members of the DND/CAF via the internet. Announcements about the survey were sent through the official DND/CAF website, organizational social networking platforms (e.g., Facebook, Twitter), and emails from senior managers and leaders. The majority of personnel learned about the survey from their chain of command (86% of Reg F, 87% of P Res, and 70% of DND PS personnel). All responses collected within 4 weeks of survey administration from April 29th, 2020, to May 22nd, 2020 were used in the present analysis.

### Sample and Demographics

A total of 27,140 DND/CAF personnel completed the survey. The current study focuses on Reg F, P Res, and DND PS personnel, which comprised 26,207 participants (96.6% of the entire sample). The majority of respondents were Reg F (*n* = 13,668; 52.2%), while a fifth were P Res members (*n* = 5,052; 19.3%), and a third were DND PS personnel (*n* = 7,487; 28.6%), which reflects the actual DND/CAF Defence Team in this regard (72).

A summary of the demographic information among the whole sample and for each specific component is presented in Table 1. Overall, the majority of the sample was male; between 25 and 44 years of age; English speaking; lived in either the National Capital Region (Ottawa/Gatineau), Ontario (outside of the National Capital Region) or Quebec (outside of the National Capital Region); were married or in a common-law relationship; and did not have any children. The majority of military respondents (Reg F and P Res) were junior or senior non-commissioned members and were not currently deployed on an operation. The most commonly reported years of service within the CAF were 1–5, 11–15, and 26 years and above. Some demographic differences were noted between components. For instance, CAF personnel (Reg F and P Res) were mostly male, while DND PS personnel were equally split into male and female respondents. Military personnel were also more likely to be under 35 years of age, whereas DND PS personnel were more likely to be between the ages of 35 and 64. P Res members were less likely than DND PS and Reg F members to be married, and DND PS members were more likely to be from the National Capital Region, while military personnel were relatively evenly distributed across Canadian regions. This too reflects the actual differences among the DND/CAF Defence Team population (71).

**Table 1:** Demographics.

	**Whole sample**	**Reg F**	**P Res**	**DND PS**
**Gender**				
Male	18,877 (72.0)	11,152 (82.0)	4,031 (80.1)	3,694 (49.7)
Female	6,926 (26.4)	2,285 (16.8)	952 (18.9)	3,689 (49.6)
Other	52 (0.3)	30 (0.2)	11 (0.2)	12 (0.2)
Prefer not to say	213 (0.8)	137 (1.0)	37 (0.7)	39 (0.5)
Missing	138 (0.5)			
**Age**				
24 years and under	3,432 (13.1)	1,644 (12.1)	1,638 (32.5)	150 (2.0)
25–34 years	6,959 (26.6)	4,719 (34.6)	1,377 (27.3)	863 (11.6)
35–44 years	6,637 (25.3)	4,114 (30.2)	777 (15.4)	1,746 (23.5)
45–54 years	5,828 (22.2)	2,552 (18.7)	828 (16.4)	2,448 (32.9)
55–64 years	3,025 (11.5)	597 (4.4)	423 (8.4)	2,005 (27.0)
65 years and over	224 (0.9)	4 (<0.1)	1 (<0.1)	219 (2.9)
Missing	102 (0.4)			
**Number of children (17 years and under)**				
0	3,408 (13.0)	1,520 (78.5)	842 (80.0)	1,046 (86.3)
1	306 (1.2)	149 (7.7)	93 (8.8)	64 (5.3)
2	247 (1.2)	124 (6.4)	67 (6.4)	56 (4.6)
3	151 (0.6)	86 (4.4)	30 (2.9)	35 (2.9)
4	49 (0.2)	32 (1.7)	11 (1.0)	6 (0.5)
5	18 (0.1)	12 (0.6)	2 (0.2)	4 (0.3)
6+	22 (0.1)	14 (0.7)	7 (0.7)	1 (0.1)
Missing	22,006 (84.0)			
**First official language**				
English	18,420 (70.3)	9,369 (69.0)	3,917 (78.0)	5,134 (69.2)
French	7,594 (29.0)	4,203 (31.0)	1,108 (22.0)	2,283 (30.8)
Missing	193 (0.7)			
**Marital status**				
Single (never married)	8,023 (30.6)	4,207 (30.9)	2,661 (52.8)	1,155 (15.6)
Separated/divorced	2,035 (7.8)	992 (7.3)	284 (5.6)	759 (10.2)
Widowed	128 (0.5)	34 (0.2)	17 (0.3)	77 (1.0)
Married/Common-law	15,907 (60.7)	8,400 (61.6)	2,076 (41.2)	5,431 (73.2)
Missing	114 (0.4)			
**Province/territory of residence**				
National Capital Region (NCR)	6,396 (24.4)	2,256 (16.5)	479 (9.5)	3,661 (49.0)
British Columbia	2,326 (8.9)	1,280 (9.4)	674 (13.4)	372 (5.0)
Alberta	1,344 (5.1)	964 (7.1)	86 (1.7)	294 (3.9)
Saskatchewan	256 (1.0)	127 (0.9)	95 (1.9)	34 (0.5)
Manitoba	897 (3.4)	560 (4.1)	174 (3.5)	163 (2.2)
Ontario (outside NCR)	5,936 (22.7)	3,497 (25.6)	1,148 (22.8)	1,291 (17.3)
Quebec (outside NCR)	4,262 (16.3)	2,336 (17.1)	907 (18.0)	1,019 (13.6)
New Brunswick	1,641 (6.3)	1,018 (7.5)	485 (9.6)	138 (1.8)
Nova Scotia	2,055 (7.8)	1,087 (8.0)	549 (10.9)	419 (5.6)
Newfoundland and Labrador	514 (2.0)	152 (1.1)	313 (6.2)	49 (0.7)
Prince Edward Island	119 (0.5)	8 (0.1)	110 (2.2)	1 (<0.1)
Northern Canada (Nunavut, Northwest Territories, Yukon)	42 (0.2)	29 (0.2)	9 (0.2)	4 (0.1)
Other (outside of Canada)	362 (1.4)	333 (2.4)	8 (0.2)	21 (0.3)
Missing	57 (0.2)			
**Rank**				
Junior NCM	9,683 (36.9)	6,622 (48.6)	3,061 (60.9)	
Senior NCM	4,102 (15.7)	3,226 (23.7)	876 (17.4)	
Junior officer	2,763 (10.5)	2,082 (15.3)	681 (13.5)	
Senior officer	2,111 (8.1)	1,700 (12.5)	411 (8.2)	
Missing	7,458 (28.8)			
**Years of service in the CAF**				
<1 year	1,403 (5.4)	512 (3.8)	434 (8.6)	457 (6.1)
1–5 years	6,570 (25.1)	2,994 (21.9)	1,958 (38.8)	1,618 (21.7)
6–10 years	3,603 (13.7)	2,254 (16.5)	726 (14.4)	623 (8.4)
11–15 years	4,646 (17.7)	2,755 (20.2)	471 (9.3)	1,420 (19.1)
16–20 years	3,203 (12.2)	1,972 (14.5)	307 (6.1)	924 (12.4)
21–25 years	2,935 (7.4)	1,201 (8.8)	266 (5.3)	468 (6.3)
26 years and over	4,776 (18.2)	1,954 (14.3)	882 (17.5)	1,940 (26.0)
Missing	71 (0.3)			

*Table presents the number of respondents (and percentage of respondents in brackets) who meet the demographic criteria. Reg F, Regular Force members; P Res, Reservists in the Primary Reserve; DND PS, Personnel in the public service of the Department of National Defence; NCM, Non-commissioned officer; CAF, Canadian Armed Forces*.

Overall, compared to population parameters of DND/CAF personnel, the resulting sample was representative of the population across key demographic variables. The only exception was a slight underrepresentation of junior non-commissioned members.

### Materials

The COVID-19 Defence Team Survey was developed by the Director General Military Personnel Research and Analysis (DGMPRA) unit of DND/CAF based on the information available about the pandemic and its potential effects on personnel, as well as consultations with organizational stakeholders (e.g., leaders and mental health experts in DND/CAF). The work of other departments within the Government of Canada was also consulted (e.g., Treasury Board Secretariat; Privy Council Office; Environment and Climate Change Canada; Innovation, Science, and Economic Development Canada) and, in particular, Statistics Canada's Canadian Perspectives Survey Series [CPSS; (45, 73–76)] and Impacts of COVID-19 on Canadians: Data Collection Series (76). Finally, COVID-19 research initiatives being carried out by allied military organizations were also considered when developing the survey.

Overall, the survey consisted of over 60 questions, five of which were open-ended questions that are the focus of this paper. These open-ended questions were used to query respondents on the following key themes: work-related challenges, personal and family-related challenges, stress management strategies, organizational support related to work, and organizational support for personal and family needs.

### Analysis of Qualitative Data

Over 75,000 responses to the five open-ended questions were obtained. A third-party coded and summarized the results following the approach delineated by DGMPRA (Human Resource Systems Group, Ltd.; see section Acknowledgments). In particular, responses for each question were coded using thematic analysis and summarized for the sample overall and for each of the components separately (i.e., Reg F, P Res, and DND PS). Some responses were complex and consisted of multiple themes. All responses were coded into all relevant themes such that complex responses that contained more than one theme were coded into each applicable theme. Given this, the total number of coded responses for each question exceeds the number of survey respondents. Responses that were deemed “Not Applicable” were removed from further analysis. It was decided to report on the seven most commonly-cited themes in response to each question, as these represented the majority of the coded themes and thus captured the most notable themes. The full set of themes in response to each question are presented in Supplementary Tables 1–5.

## Results

### Work-Related Challenges

**Figure 1:**
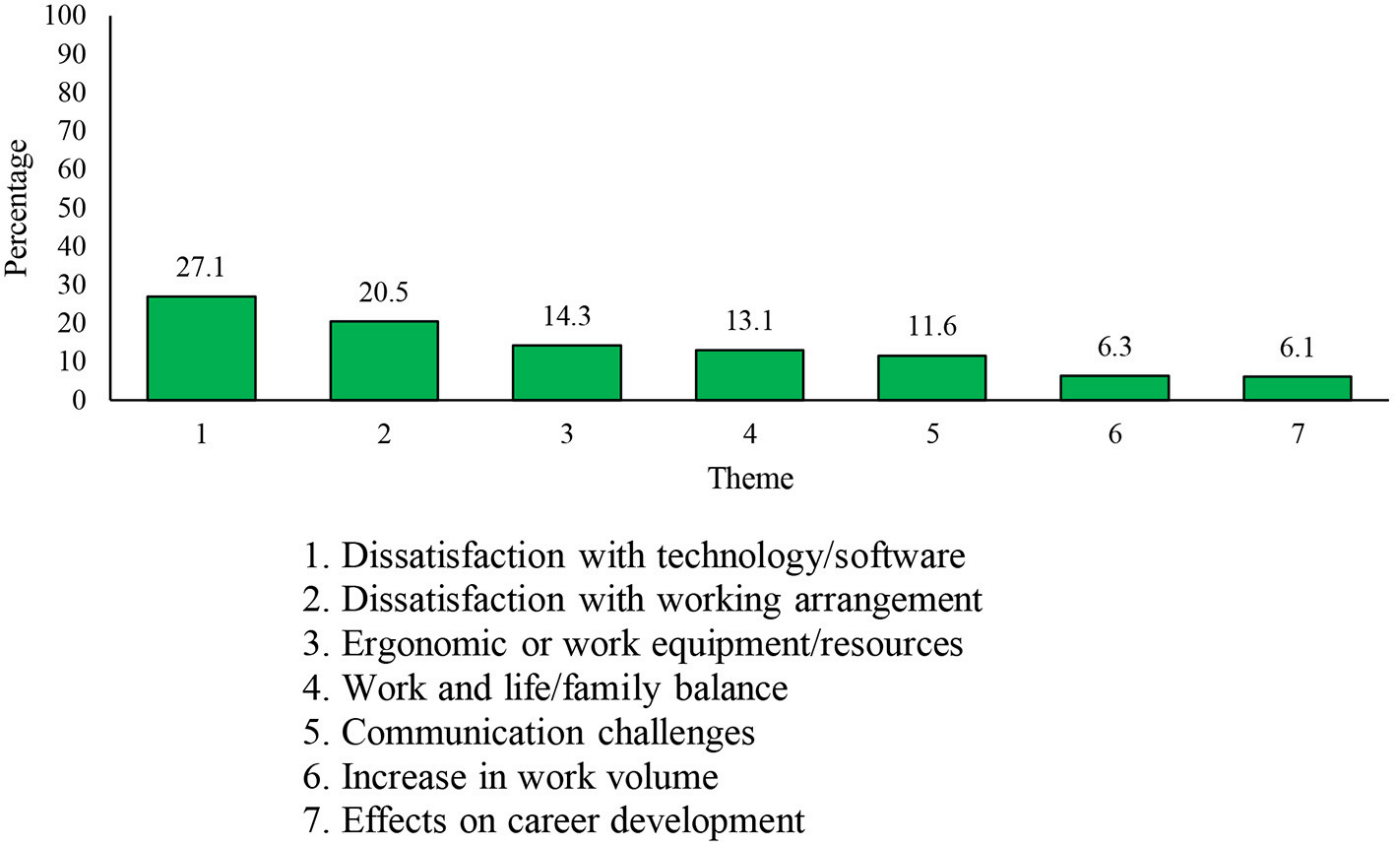
Seven most frequently mentioned work-related challenges.

The first open-ended question asked respondents, “What are the most significant work-related challenges you have been experiencing since the start of the COVID-19 pandemic?” A total of 19,139 respondents (9,173 Reg F, 3,998 P Res, and 5,968 DND PS members) responded to this question. Twenty-nine themes (Supplementary Table 1) were extracted from statements, and 522 statements were deemed “Not Applicable.” The seven most common themes in response to this question included (1) *dissatisfaction with technology/software*, (2) *dissatisfaction with one's working arrangement*, (3) *ergonomic or work equipment/resources*, (4) *work and life/family balance concerns*, (5) *communication challenges*, (6) *increases in work volume*, and (7) *effects on career development* (see Figure 1).

The first three themes focused on material limitations to establish an appropriate workspace in the home and successfully work from home. The most commonly-reported theme was *dissatisfaction with technology/software* and was reported by *n* = 5,038 (27.1%) respondents. Responses in this theme centered on challenges in accessing work-related email and software, connecting to the organization's virtual private network (VPN), and insufficient IT support. The second most common theme was *dissatisfaction with working arrangements* (*n* = 3,810, 20.5% of respondents), including challenges related to the transition to working from home, a perceived lack of productivity, lack of motivation, disruptions in routine, distractions making it difficult to focus on work, and requirements for new and unknown skills to successfully work from home. For instance, one respondent noted that “It takes days to do what could be done in minutes at the office.” The third most common theme was *ergonomics of work equipment/resources* (*n* = 2,659, 14.3% of respondents), which entailed respondents' physical or functional discomfort of their new work environment due to concerns, such as insufficient office space, office furniture, and hardware.

The fourth most common theme highlighted disruptions in *work and life/family balance* (*n* = 2,439, 13.1% of respondents). Comments within this theme highlighted the challenge of balancing work-related responsibilities, home/childcare responsibilities, and the need for leisure, entertainment, and relaxation. Respondents also noted that these difficulties directly resulted from working while confined to their home with their family, and that they were experiencing a toll on their well-being due to a lack of separation of work and personal domains (e.g., having to be constantly accessible for work).

The fifth most common theme was *communication challenges* (*n* = 2,155, 11.6% of respondents), highlighting dissatisfaction with various aspects of work-related online communication, rules and regulations relating to the ongoing pandemic, and the organization's decisions to address the ever-evolving situation of the COVID-19 pandemic. This theme also included reports on difficulties contacting peers, subordinates, and supervisors, and delayed responses from peers and supervisors as a result of being limited to online rather than in-person interactions.

The sixth theme was an *increase in work volume* (*n* = 1,177, 6.3% of respondents), and included comments related to increases in work volume, reduced staff/manning, new tasks or demands stemming from the pandemic, and a generally high work volume. Some of the comments coded within this theme pertained to the respondents' reduced capacity while working from home, difficulties getting work done in time, burnout resulting from greater workloads, and difficulties delegating work.

The seventh most reported theme was *effects on career development* (*n* = 1,144, 6.1% of respondents). Responses categorized in this theme related to a variety of concerns regarding career development, such as lack of recognition for increased work or tasks, uncertainty about the respondent's training and education, and general concerns about career progression and promotion.

**Figure 2:**
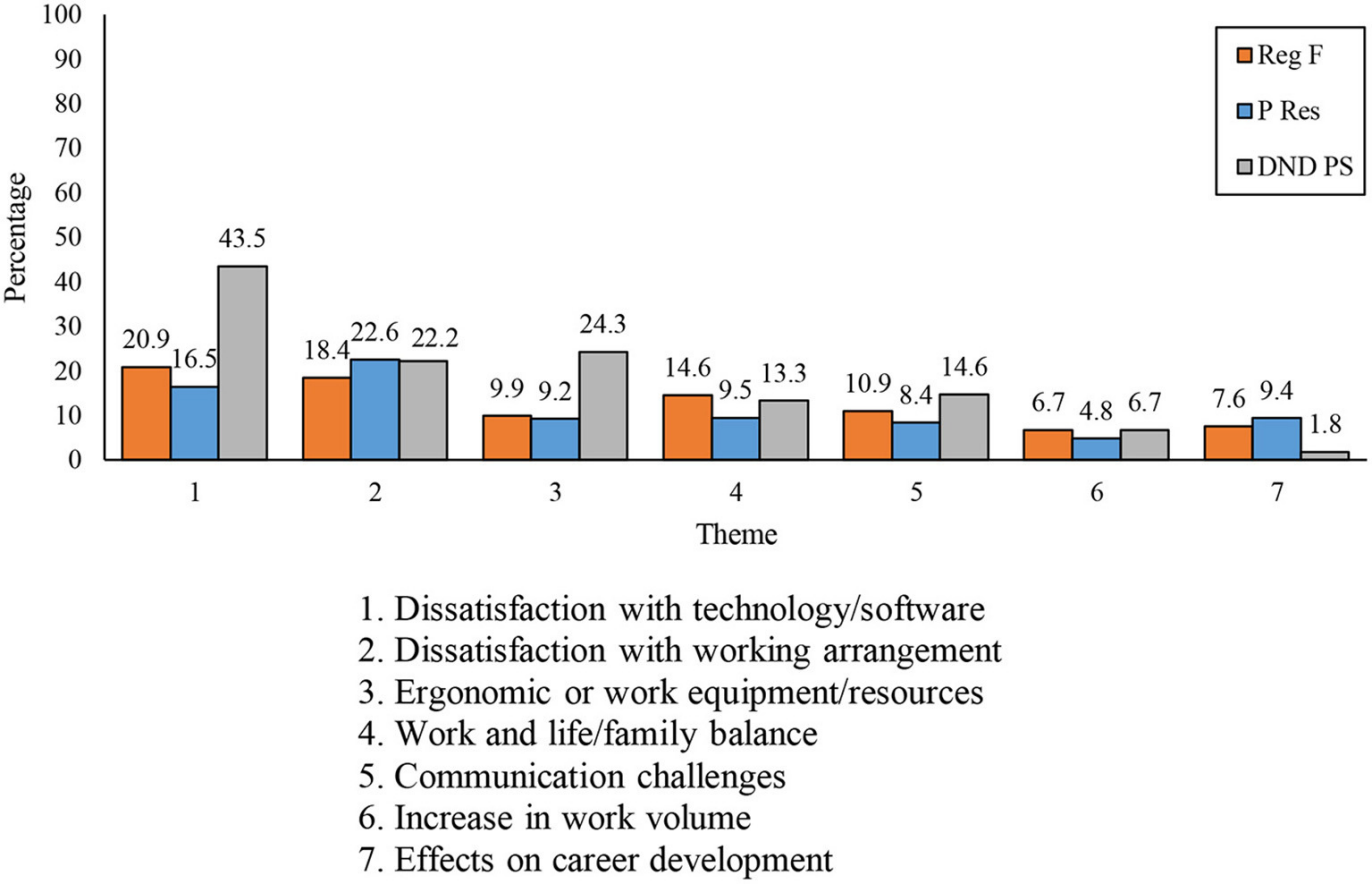
Seven most frequently mentioned work-related challenges by component.

The most notable differences observed between components, as shown in Figure 2, were that DND PS members were substantially more likely to report *dissatisfaction with technology/software*, issues relating to *ergonomic or work equipment/resources*, and were somewhat more likely to report *communication challenges*. Military members on the other hand, including those from the P Res and the Reg F, were more likely to report concerns of *career development*.

### Personal and Family-Related Challenges

**Figure 3:**
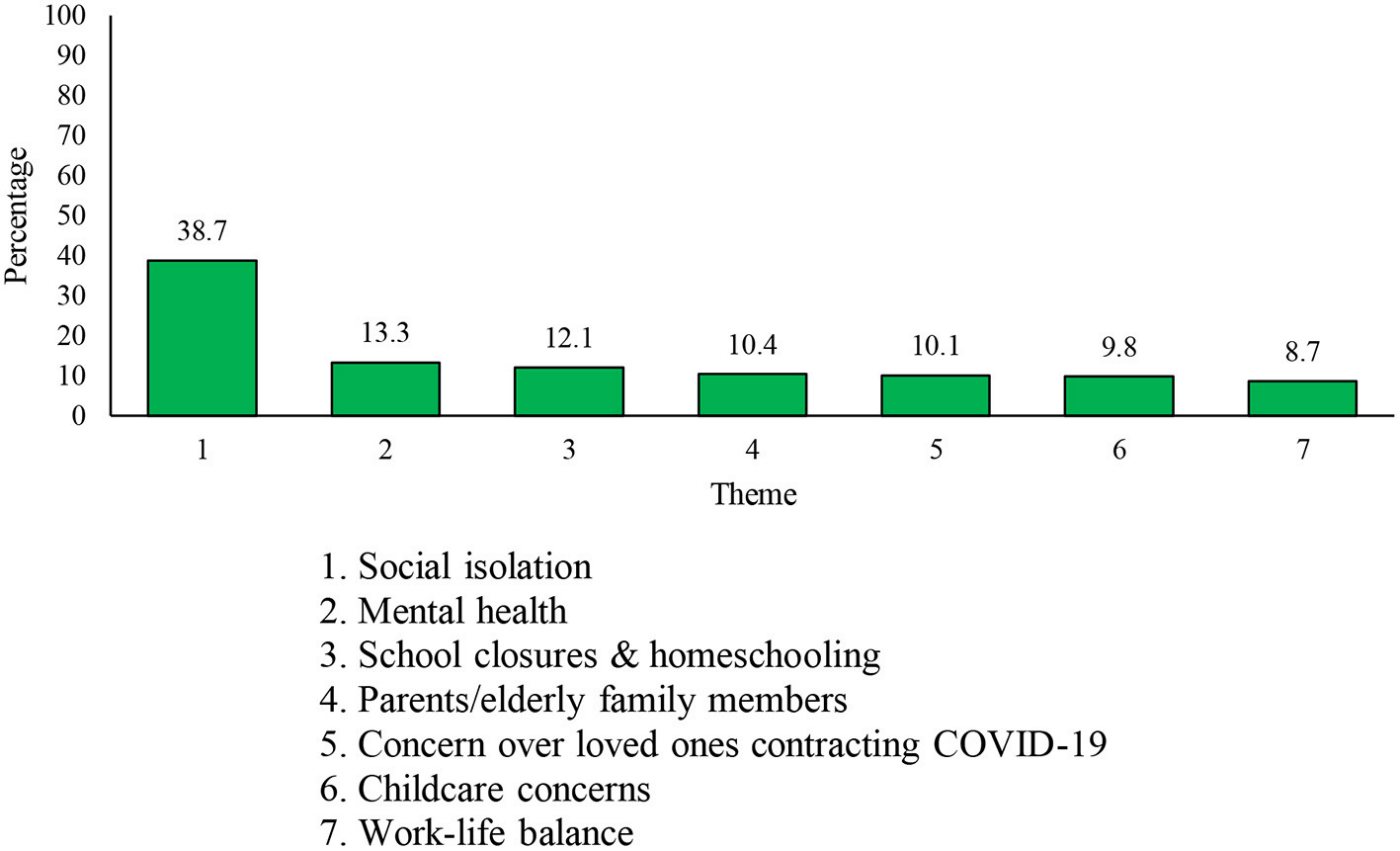
Seven most frequently mentioned personal and family-related challenges.

The second open-ended question asked respondents “What are the most significant personal and family-related challenges you have been experiencing since the start of the COVID-19 pandemic?” A total of 18,544 respondents (9,006 Reg F, 3,790 P Res, and 5,748 DND PS members) provided an answer to this item. Two-hundred and thirty-eight responses were coded as “Not Applicable.” Thirty-one themes were coded from valid responses to this question, with the seven most common being (1) *social isolation*, (2) *mental health*, (3) *school closures and homeschooling*, (4) *parents/elderly family members*, (5) *loved-ones contracting COVID-19*, (6) *childcare concerns*, and (7) *work-life balance* (see Figure 3).

The *social isolation* theme (*n* = 7,089, 38.7% of respondents) was the most cited theme and included comments related to limited gatherings with family members, friends, and co-workers; a general lack of social gatherings; lack of public entertainment and social events; and an inability to travel. Sample comments include “Missing the human contact and interaction at work” or “Unable to reunite with my partner who lives outside of the (National Capital Region).” Some comments also included the emotional toll that these restrictions were taking, such as increased loneliness (e.g., “Living alone, I'm very lonely!”).

The second most common theme in response to this question related to respondents' *mental health* (*n* = 2,438, 13.3% of respondents). Respondents mentioned several negative consequences the pandemic and social changes had on their mental health and well-being. Such consequences included increases in anxiety, depression, irritability, impatience, burnout, boredom, and difficulties remaining motivated to work.

Two themes highlighted the challenge and stress of having to balance work and childcare during the pandemic. The third most common theme in response to this question was *school closures and homeschooling* (*n* = 2,208, 12.1% of respondents), which included comments noting the difficulty balancing childcare and work, carrying out homeschooling effectively, and ensuring quality education for the respondents' child(ren). Respondents also commented on the stress and mental health toll of this added challenge (e.g., “Homeschooling my three young children causes me no end of stress”). Moreover, homeschooling was discussed as particularly challenging when considering children's individual dispositions or needs, such as learning disabilities. Relatedly, in the sixth most common theme, respondents reported *childcare concerns* (*n* = 1,792, 9.8% of respondents), such as concerns regarding the financial obligations of childcare, the inaccessibility of regular and emergency childcare, and lack of support for children with special needs.

The fourth most common theme evinced concerns for *parents/elderly family members* (*n* =1,904, 10.4% of respondents), which consisted of comments noting stress and concern for family members, the need to provide care and support to medically vulnerable and elderly family members, and worrying about their susceptibility to COVID-19 complications (e.g., “My parents are in the age category that puts them at risk. I am concerned for their well-being”). Some respondents also reported stress and frustration with ensuring the compliance of their family members with COVID-19 restrictions. For instance, one respondent noted “I am having to manage my parents, to make sure they don't go out, and making sure they have what they need.” Another noted that “Trying to explain why social distancing and isolation is required with older family members that don't understand it.”

Relatedly, the fifth most common theme highlighted anxieties regarding the health of loved ones amid the severity of the pandemic. This theme, *concern over loved one contracting COVID-19* (*n* = 1,848, 10.1% of respondents), included comments centering on the fear of a close loved one contracting the virus, the vulnerability of loved ones working as essential workers and first-responders in compromised spaces, and the risks posed by such circumstances to vulnerable family members. For example, one respondent noted “Fearing for the safety of my spouse who is a nurse who has been working (with) COVID patients.”

The seventh most common theme was *work-life balance* (*n* = 1,590, 8.7% of respondents) which mirrored the *work and life/family balance* theme extracted when surveyed on work-related challenges (see section Work-Related Challenges). Responses in this theme further highlighted how the increasing demands of working from home, changes in workload, and teleworking have encroached on one's non-work-related endeavors. Many also added that a disrupted work-life balance is contributing to burnout: “Before the pandemic, work stayed at work. Now it is at home. Using my own emails and devices due to departmental challenges has been extremely intrusive to maintaining a work life balance.” Another respondent noted “Due to network issues, I've had to change my work hours to be in the evening as well. This means that I am working on and off over a 16-h time period and my personal time is suffering and I'm not able to dedicate much time to my life outside of work.”

**Figure 4:**
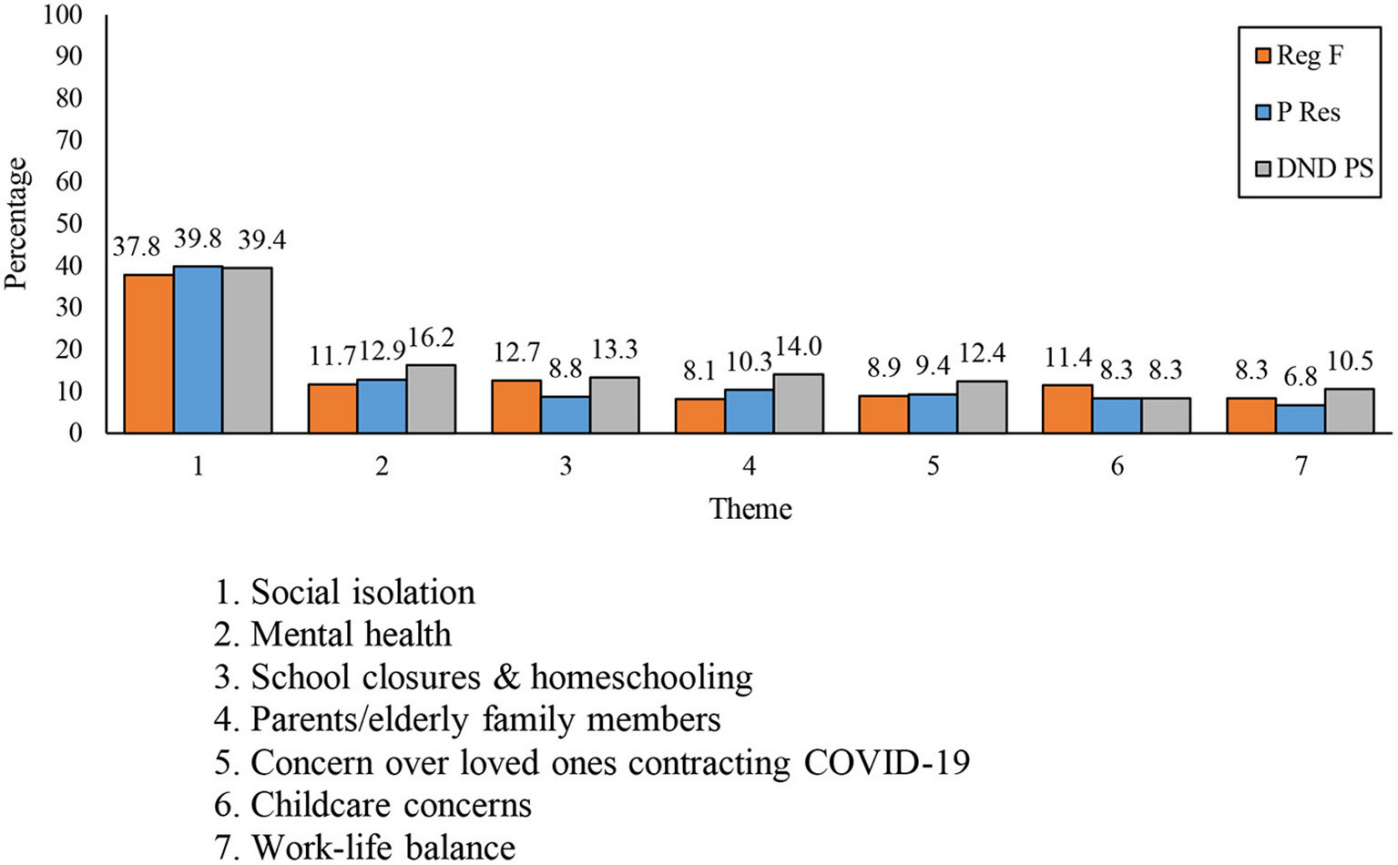
Differences between components of respondent in the frequency of the seven themes mentioned.

There were no particularly notable differences in terms of personal and family-related challenges among Reg F, P Res, and DND PS respondents (see Figure 4).

### Stress Management Strategies

**Figure 5:**
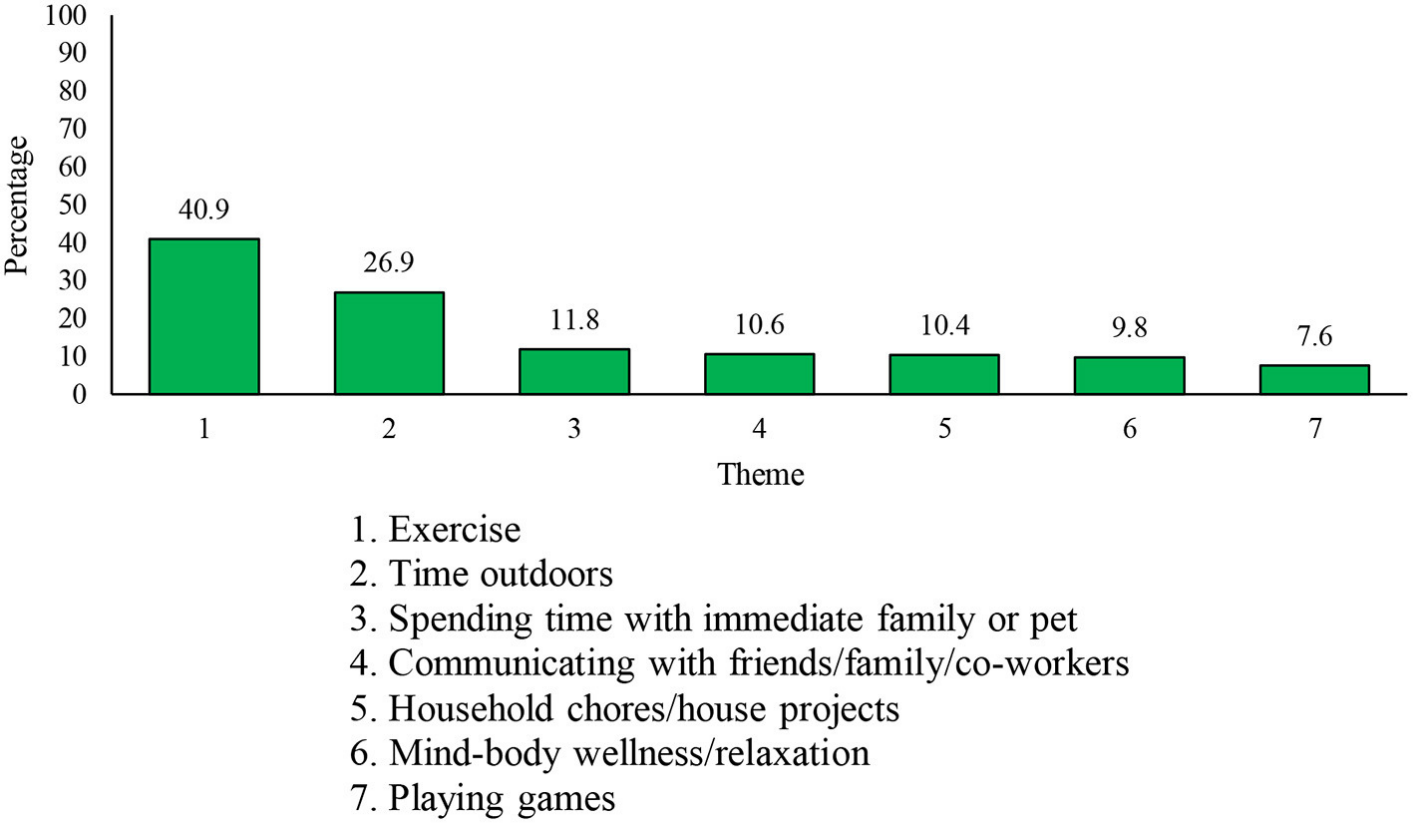
Seven most frequently mentioned stress management strategies.

The third open-ended question asked respondents, “What stress management strategy(ies) have you found most helpful to get you through the COVID-19 pandemic?” A total of 17,826 respondents (8,692 Reg F, 3,881 P Res, and 5,253 DND PS) answered this question. One-hundred and seventy-four responses were deemed “Not Applicable.” Thirty-eight themes were derived, with the seven most common being (1) *exercise*, (2) *time outdoors*, (3) *spending time with immediate family or pet*, (4) *communicating with friends/family/coworkers*, (5) *household chores/house projects*, (6) *mind-body wellness/relaxation*, and (7) *playing games* (see Figure 5). To note, while relatively infrequent (representing < 2% of responses), some of the coping strategies reported may be considered maladaptive, such as drinking alcohol (*n* = 259, 1.5%), cannabis use (*n* = 163, 0.9%), and smoking cigarettes and/or cigars (*n* = 44, 0.2%).

The most commonly reported stress management strategy was *exercise* (*n* = 7,125, 40.9% of respondents), which included any form of physical exercise completed either indoors or outdoors. The second most common strategy, *time outdoors* (*n* = 4,750, 26.9% of respondents), constituted references to spending time outdoors doing a range of activities (e.g., getting fresh air, gardening/yard work, exercise outdoors, being in the sun/nature). The third strategy, *spending time with immediate family or pet* (*n* = 2,084, 11.8% of respondents) included establishing and maintaining quality time with family or pets. The fourth most common strategy, *communicating with friends/family/coworkers* (*n* = 1,865, 10.6% of respondents), included using technological mediums to connect with others for work or leisure. Completing *household chores/house projects*, including home renovations, was mentioned by *n* = 1,843 respondents (10.4% of respondents). *Mind-body wellness/relaxation* strategies (*n* = 1,723, 9.8% of respondents) was the sixth most commonly-reported coping strategy and consisted of practices, such as relaxation and breathing techniques, yoga, meditation, and mindfulness. The seventh most common strategy was *playing games* (*n* = 1,340, 7.6% of respondents), which included completing puzzles and playing board games and video games.

**Figure 6:**
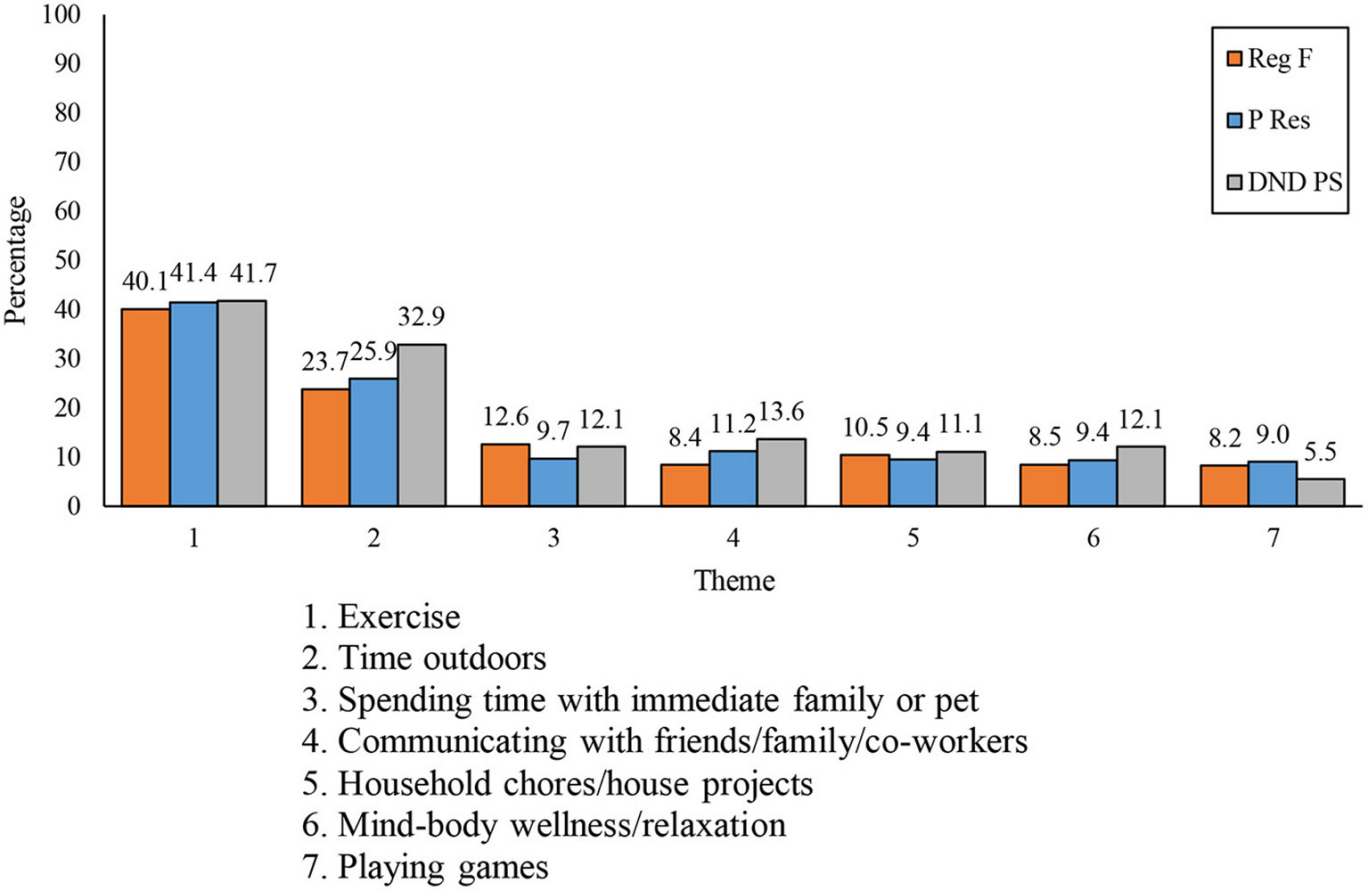
Seven most frequently mentioned coping strategies among each component.

The use of these coping strategies was generally similar across personnel from the three components (see Figure 6), although DND PS respondents were slightly more likely to indicate spending time outdoors and slightly less likely to indicate playing games as coping strategies.

### Work-Related Organizational Support

**Figure 7:**
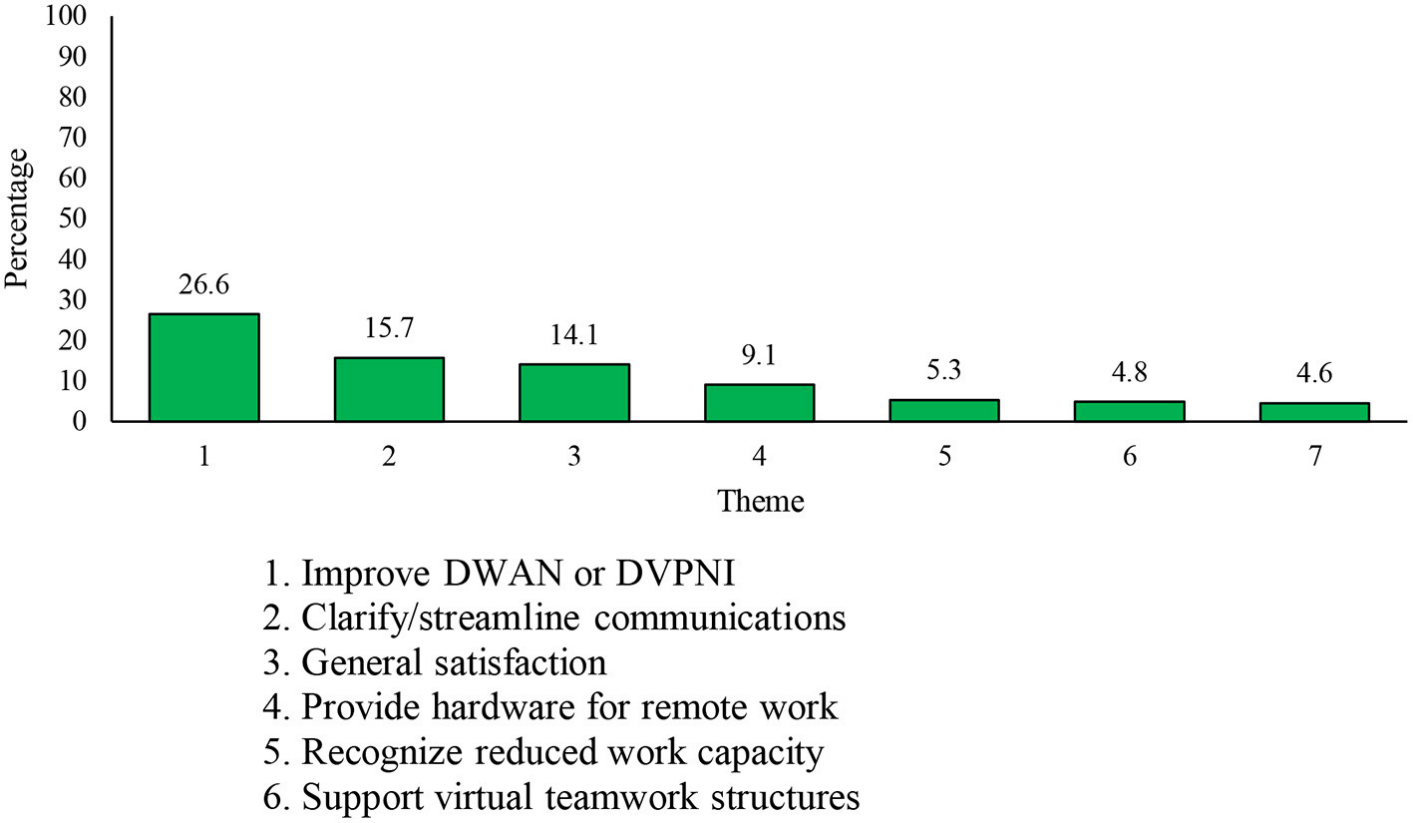
Seven most frequently mentioned themes relating to how the DND/CAF can better support work during the COVID-19 pandemic.

The fourth open-ended question asked respondents “What can the DND/CAF do to better support you with your work during the COVID-19 pandemic?” A total of 9,994 respondents (4,846 Reg F, 1,895 P Res, and 3,253 DND PS) answered this question. Two-hundred and seventy-four responses were deemed “Not Applicable.” Thirty-one themes were derived from responses, the seven most common being (1) *improve IT network*, (2) *clarify/streamline communications*, (3) *general satisfaction*, (4) *provide hardware for remote work*, (5) *recognize reduced work capacity*, (6) *support virtual teamwork structures*, and (7) *flexibility for work location/hours* (see Figure 7). The *general satisfaction* theme (*n* = 2,582, 14.1% of respondents) included comments indicating that the respondent was satisfied with the DND/CAF's efforts to support their work, and most of the comments within this theme (98.3%; *n* = 1,348) did not offer elaboration regarding their satisfaction.

Overall, the most commonly-cited recommendation was to *improve IT network* (*n* = 2,582, 26.6% of respondents). Comments comprising this theme related to the need to rapidly improve the online infrastructure for personnel to access their work-related resources available only via a secured VPN connection. Comments within this theme also highlighted the need to improve network accessibility and bandwidth capacity. To note, some respondents added that the limitations in bandwidth have necessitated that they work outside of their regular work hours, including early in the morning or late into the evening. Respondents also suggested the value of centralized and consistent login credentials, and better integration, across networks within the organization. Finally, respondents suggested the use of rotating shifts or work schedules to improve accessibility to the network.

The second most common recommendation was to *clarify/streamline communications* (*n* = 1,527, 15.7% of respondents) to ensure easy access to messages and communications for all personnel. Respondents noted that, at the time of the survey, they were oversaturated with information from multiple sources (some of which consisted of conflicting information across sources). Respondents also mentioned that they would appreciate a reduction in communications that they deemed unnecessary and/or abstract. For example, one respondent noted “Streamline communications. There is too much information on too many platforms. I rely on my work email and cell phone for communications normally, but now I have to monitor Zoom, Slack, Google Docs, and my personal email to stay up to date on everything” and “Stop pushing multiple department wide messages and policies that tend to overwhelm the in-box and, because they are departmentally focused, are generally so ambiguous as to mean little or nothing to the individual at the tactical level. DND wide traffic and messages should be focused on concise, brief, highly important or urgent messages.”

The fourth most common theme (*n* = 886; 9.1% of respondents) entailed recommendations that the organization *provide hardware for remote work*. Comments within this theme highlighted a lack of technological, computational, and communication equipment to complete work from home. Provision of ergonomic equipment and office furniture was also recommended. Similarly, the sixth most common theme was labeled *support virtual teamwork structures* (*n* = 469, 4.8% of respondents), which entailed the provision of additional software, IT support, and training to operate virtual team structures (e.g., Microsoft Teams).

The remaining themes entailed ways in which supervisors, senior leaders, and the organization can offer flexibility and understanding to personnel as they cope with the pandemic. In particular, the fifth most common theme, *recognize reduced work capacity* (*n* = 519, 5.3% of respondents), focused on the need for supervisors and leaders to recognize the difficulties of working from home, and working while caring for children or supporting other family members. Comments also included the notion that this understanding should be applied to performance evaluations. Relatedly, the seventh most commonly cited theme, *flexibility for work locations/hours* (*n* = 447, 4.6% of respondents), related to the need for greater autonomy in return-to-work decisions, flexibility in work hours, and flexibility to accommodate frequently changing and challenging home situations.

**Figure 8:**
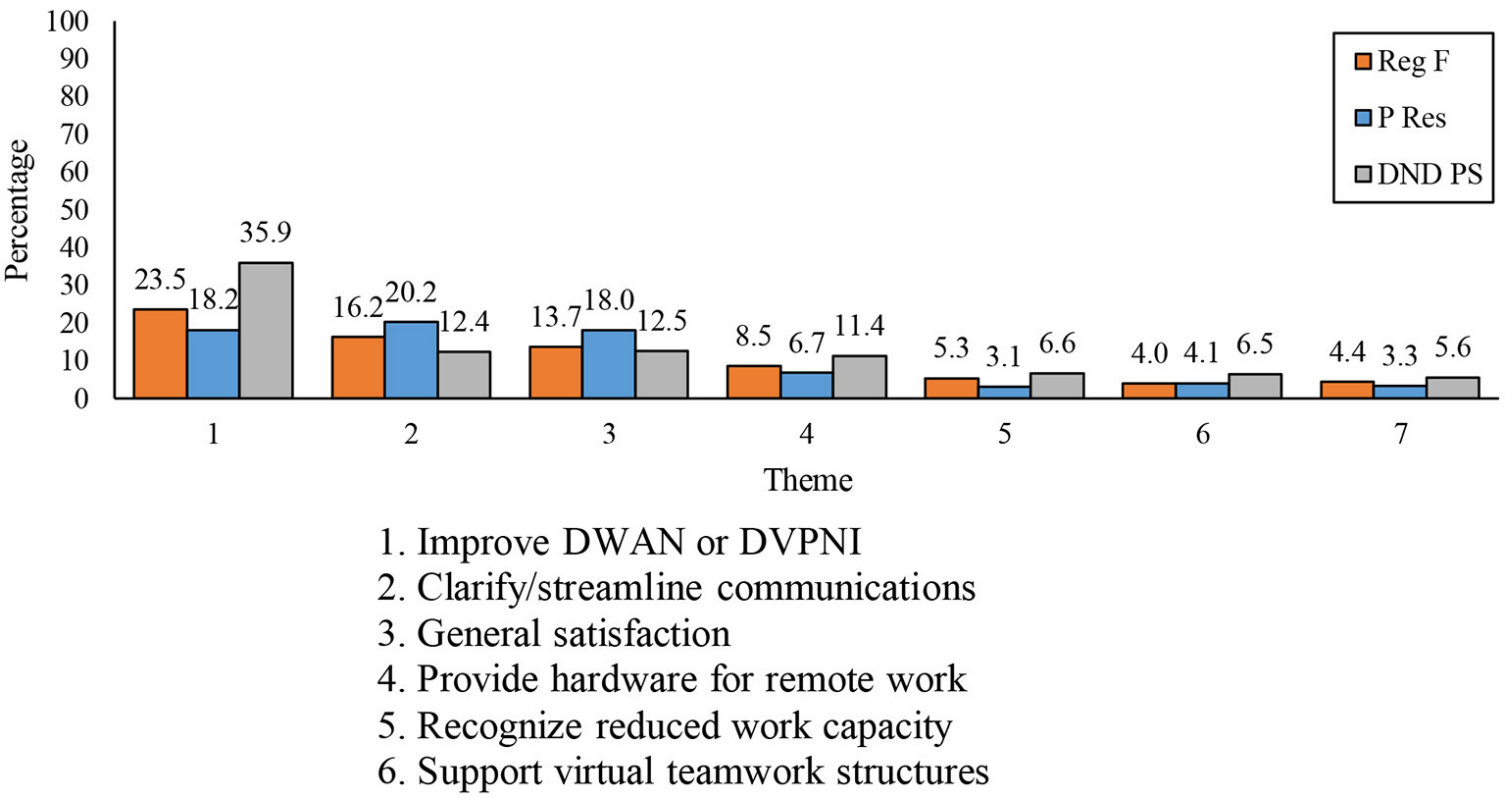
Differences between components in terms of the seven most commonly reported themes on changes the organization could make to support its members' work.

Few differences were observed among the components (see Figure 8). DND PS personnel were more likely to mention the need to *improve DWAN or DVPNI*, whereas P Res members were the most likely to mention the need for improving communications.

### Organizational Support for Personal and Family Needs

**Figure 9:**
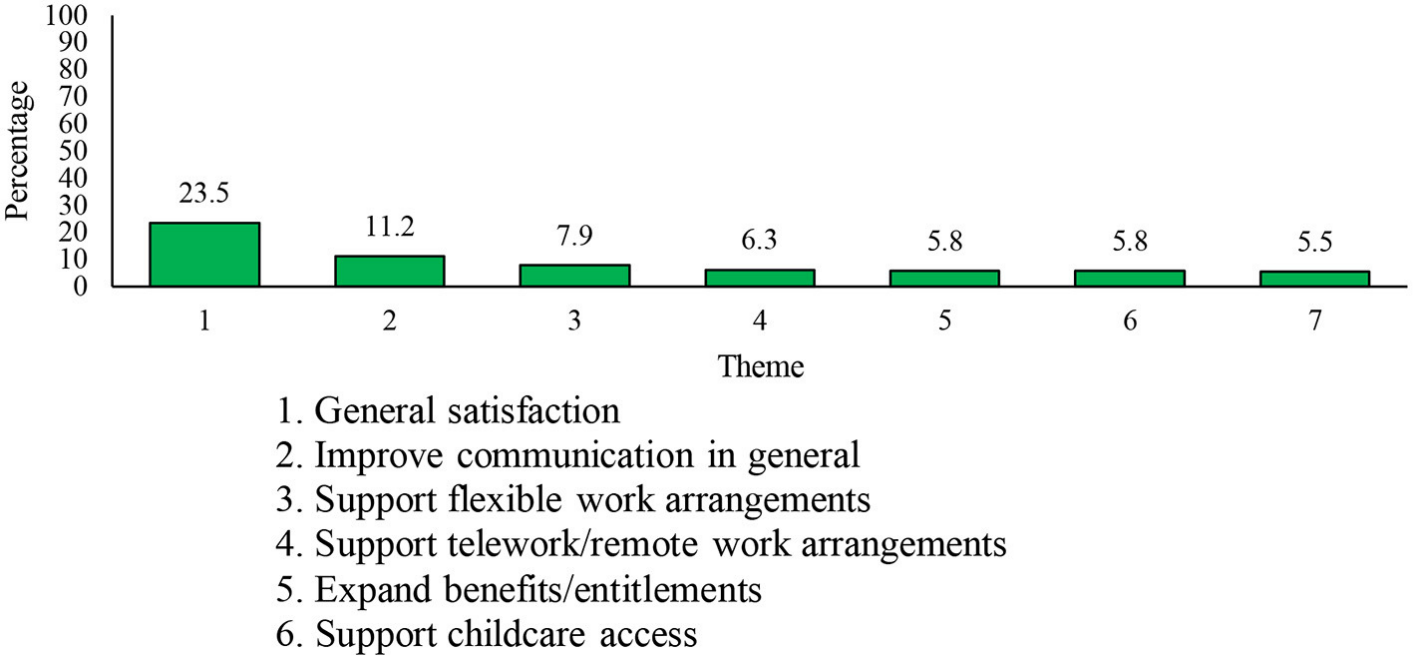
Seven most frequently mentioned themes relating to how the DND/CAF can better support personal and family needs during the COVID-19 pandemic.

The fifth open-ended question asked respondents “What can the DND/CAF do to better support you with your personal and family needs during the COVID-19 pandemic?” A total of *n* = 7,105 respondents (3,729 Reg F, 1,232 P Res, and 2,144 DND PS) answered this question. Three-hundred and ten responses were deemed “Not Applicable.” Thirty-one themes were derived from comments (see Figure 9), the seven most common being (1) *general satisfaction*, (2) *improve communication in general*, (3) *support flexible work arrangements*, (4) *support telework/remote work arrangements*, (5) *expand benefits/entitlements*, (6) *support childcare access*, and (7) *consideration for childcare and homeschooling*. The *general satisfaction* theme (*n* = 1,597, 23.5% of respondents) included comments indicating contentment with DND/CAF's efforts to support their personnel's personal and family-related well-being amid the COVID-19 pandemic. Most responses (96.2%; *n* = 1,537) that contained a comment coded into “*general satisfaction*” did not contain further comments (i.e., respondents were generally satisfied and had no further suggestions).

The three most common recommendations for personal support mirrored recommendations provided in response to the previous question (section Work-Related Organizational Support). The most common recommendation was to *improve communication in general* (*n* = 760, 11.2% of respondents), which highlighted a need to increase communication lines from senior management/leaders, centralizing and prioritizing communications, and better communication regarding services available to personnel. The second most common recommendation was that leaders *support flexible work arrangements* (*n* = 536, 7.9% of respondents). Comments within this theme related to a desire for greater autonomy and flexibility to decide upon work routines [e.g., allowing individuals leave during regular work hours to handle essential needs (e.g., groceries)], a reduction in work-related activities and meetings, and providing flexibility on return-to-work decisions. The third most common area of support was related to support for telework/remote work arrangements (*n* = 426, 6.3% of respondents), which constituted an expansion of telework roles, providing guidance and training for long-term telework, and increasing support and hardware for telework.

The remaining recommended areas of support were unique to the current question. In particular, the fourth most common recommendation was to *expand benefits/entitlements* (*n* = 397, 5.8% of respondents), including the need to expand and communicate the financial resources available to personnel. In a few cases, this included expanding the definition of supported family and/or dependents to include elderly parents and/or extended family members. Members also desired an expansion of services provided by DND/CAF, notably the inclusion of psychosocial services (i.e., social work).

The remaining two themes, which centered on support for those engaging in home schooling and extended childcare (*n* = 394; 5.8% of respondents), recommended the organization support childcare access. This included an expansion of the services provided by the CAF Military Family Resource Centers, financial assistance for childcare, access to emergency medical childcare, and the implementation of flexible hours to accommodate childcare. Another 371 (5.5%) respondents commented on homeschooling in particular, and recommended reducing the workload for members who were homeschooling children, providing flexibility to workers to accommodate homeschooling responsibilities, and offering appropriate guidance for caring for children and managing homeschooling while working from home.

**Figure 10:**
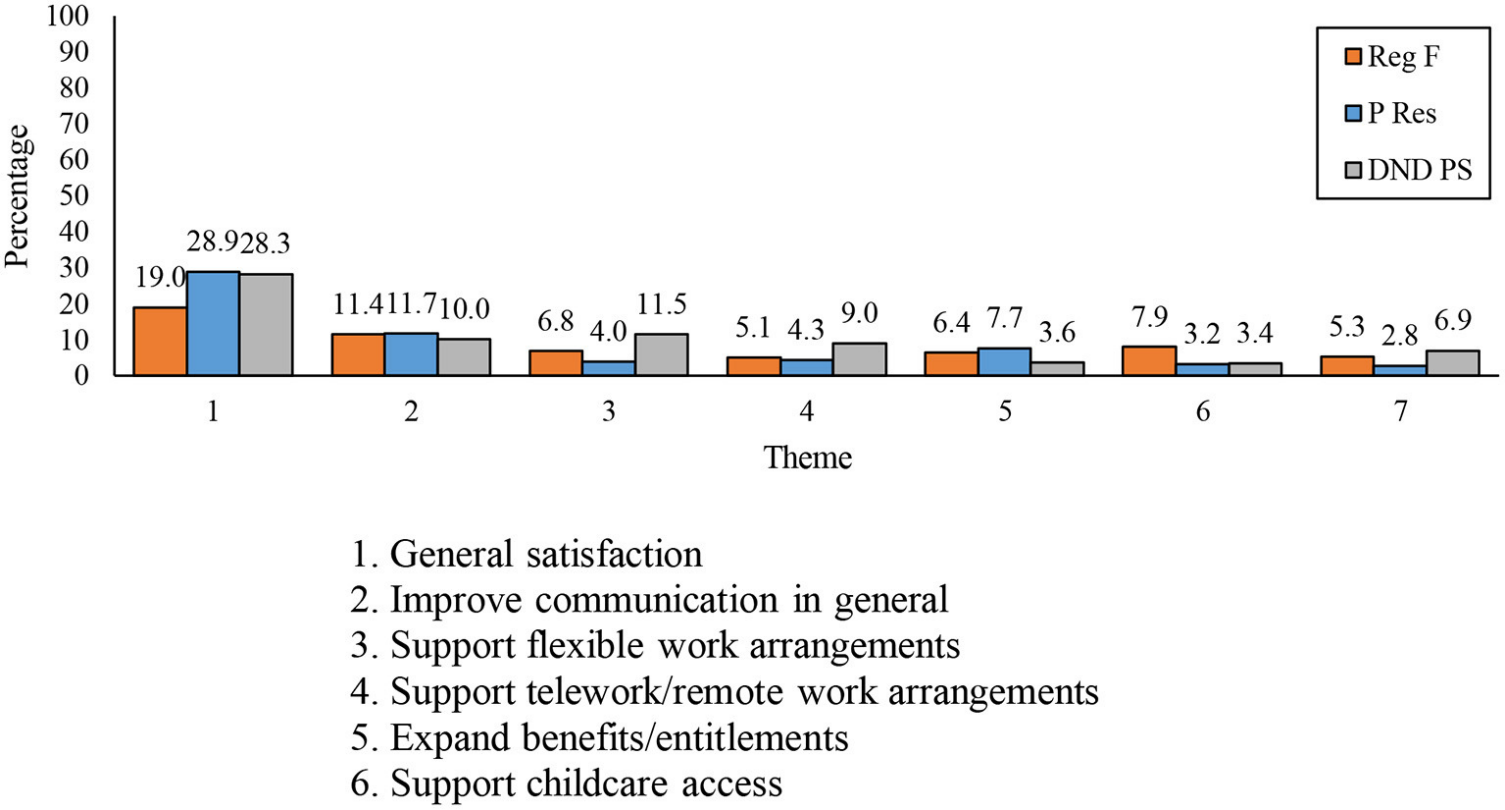
Seven most frequently mentioned themes relating to how the DND/CAF can better support personal and family needs during the COVID-19 pandemic, by component.

With respect to differences by component, members from the P Res and DND PS were more likely to note being generally satisfied with the organizational support provided as compared to their Reg F counterparts (see Figure 10). By contrast, Reg F members were more likely than P Res and DND respondents to mention childcare access and expansion of benefits and entitlements as areas with which the organization can offer more support.

## Discussion

The COVID-19 Defence Team Survey was developed to provide insight on the challenges faced by civilian and military personnel in the wake of the COVID-19 pandemic and what the DND/CAF organization can do to support its personnel. Considering working arrangements were drastically altered with little notice, work-related and personal/family-related well-being were key areas examined. Moreover, given that the effects of the pandemic may differ depending on individual characteristics (15, 22, 28), and in light of the distinct roles and employment arrangements of Reg F, P Res, and DND personnel, the experiences and concerns of personnel from these three DND/CAF components were compared. In brief, a common aspect of many of the most commonly reported challenges—on both the personal and work front—is that these touch on several aspects of respondents' experiences with having to adjust to working from home, with little time to prepare on both their part or the part of DND/CAF.

### Work-Related Challenges

The most commonly reported work-related challenges during the pandemic centered on issues with technology when working from home; lack of proper workspace, resources, and ergonomic equipment; work/family balance concerns; increased communication difficulties; increases in work volume; and concerns around career development. In addition to being consistent with findings reported in other Canadian (9, 28) and international (29–34) surveys, these findings are in line with pre-pandemic research on telework/working from home. For instance, dissatisfaction with technology was a common concern that resulted in lost work time and frustration (34, 77). In the current study, respondents' dissatisfaction with technology was accompanied by reports that their work required much more time and effort and thereby negatively affected their productivity. Pre-pandemic research also demonstrated ergonomic challenges among workers transitioning to a telework/work from home environment, which contributed to poor posture, neck pain, sore eyes, fatigue, work-oriented discomfort, and increased employee expenses (34, 78). Individuals reported similar issues in the current study, which most likely resulted from the fact that most were unprepared for working from home and did not have adequate space or equipment at their disposal to create an ergonomic home work environment. These reported difficulties are likely to increase job strain (78), and result in other mental health concerns (79–82) if left unaddressed.

Other work-related issues, including higher workloads and concerns with career progression, may have been more unique to the current pandemic. Greater workload may have resulted from an increase in specific duties related to the pandemic, difficulty accomplishing one's work in light of inadequate technology or equipment, or even from employees' personal sense of duty to allocate time that was previously dedicated to commuting toward their work (30, 40). Increasing workloads have been found to overwhelm personnel and exacerbate feelings of loneliness, emotional exhaustion, and the frequency of miscommunication within the organization (83).

While military personnel in the CAF had previously noted concerns related to their career progression (84), our results suggest that these concerns may have been exacerbated by telework in the context of the pandemic. In particular, Reg F and P Res respondents reported that the pandemic fostered uncertainty around obtaining training and education required for their career and a lack of clarity about their development and career progression. Past research has shown that perceived barriers or plateau in career progression can lead to a reduced job satisfaction, negative affect and feelings of injustice from workers directed toward the organization's leaders, a lack of work motivation, and dissociation between the organization's and the worker's goals and values (85–89).

Finally, many respondents highlighted communication challenges in the context of the current pandemic. Past CAF research has demonstrated that both the quality and quantity of information received from leaders is correlated with job satisfaction and commitment to the organization (90). Work-related organizational communication has also been shown to impact retention in both military (84, 91) and civilian organizations (92, 93). Moreover, dissemination of conflicting information from multiple sources can be particularly dangerous when considering the risks of COVID-19. Such messaging can be overwhelming for members and lead to disregarding important information about prevention. Indeed, information overload has been found to be associated with an increased fear of contracting COVID-19, as well as less vigilance regarding the dangers of COVID-19 (i.e., lower likelihood of self-isolating or physical distancing) and increased sharing of misinformation about the virus (94–96). Clear, accurate, timely, and streamlined communication is critical to provide personnel with information on how to better prepare and protect themselves during the pandemic, and to provide them with key updates about organizational changes or directives and relevant programs, services, and other resources designed to support them.

### Personal Challenges

Personnel also reported a range of personal challenges. Work-life balance concerns (also reported as a work-related challenge in the present study), may have resulted from several factors, such as a high workload and a lack of separation between home and work life (84, 97). Respondents also reported other personal challenges that may have contributed to dissatisfaction with one's work-life balance, including increased parenting demands due to loss of access to childcare, limited childcare resources and support, and school closures. Another main personal concern stemming from the pandemic was worry and stress related to the well-being of family members, especially those particularly vulnerable to the COVID-19 virus. As a result, personal time was often dominated by caring for family at the expense of time previously available for leisure, relaxation, and/or entertainment (98). Work-life imbalance can have a substantial impact not only on individuals' well-being, but also on personnel retention. For instance, retention surveys of military personnel in the CAF, the Australian Defence Force, and the New Zealand Defence Force have demonstrated that 40–50% of members reported dissatisfaction with work-life balance, which was a key element in decisions to leave the military (84, 97).

Personnel also reported that their mental health has suffered as a result of the pandemic, which is consistent with studies on the mental health outcomes of the pandemic among civilians in Canada (41, 45–47) and abroad (42, 43), and among military personnel (15, 64). Respondents in this study reported feeling anxiety, burnout, and depression due to balancing the increasing demands of their home and work lives; loneliness and sadness due to social isolation; and anxiety and emotional exhaustion regarding the dangers of the COVID-19 pandemic and its future outcomes. Research has identified a wide variety of strategies used by individuals to cope with these mental health challenges, including maintaining a positive outlook, remaining busy, connecting to one's religion, communicating with others, and physical activity (99, 100). In the current study, personnel reported using a wide range of coping strategies, including physical exercise, meditation and mindfulness, spending time and communicating with family, and keeping busy with household projects. Indeed, these may be effective coping strategies to help ameliorate the impact of the COVID-19 pandemic, as the adoption of such strategies has been associated with reductions in anxiety and depression and better mental health outcomes in other research (100–102).

Some personnel reported arguably negative coping approaches, such as increased use of cannabis, nicotine, and alcohol. Indeed, research shows that some individuals have been struggling to adopt effective strategies to cope with stress in the face of the COVID-19 pandemic (99). It would be beneficial for the organization to offer resources and training related to effective coping in order to support the health and well-being of its members as the pandemic evolves.

### Distinctions Between Components of Personnel

Given that the pandemic may affect different groups of personnel differently, its impacts on three broad groups of personnel in the DND/CAF, including Reg F, P Res, and DND PS personnel, were compared. Overall, members from the three broad groups shared similar concerns, though there were a few noteworthy differences. Specifically, DND PS personnel were more likely to report technological and ergonomic issues relative to Reg F and P Res personnel. This finding is understandable, given that DND PS members were more likely to be working from home relative to Reg F and P Res members (15). On the other hand, P Res personnel were the most likely to voice concerns regarding communication. This is not entirely surprising given that insufficient communication has been a long-standing issue for Canadian reservists (66, 103). Such challenges may be connected to the fact that reservists often have infrequent access to the departmental network and, consequently, fewer opportunities to interact with their military chain of command. This may especially hold true for reservists who are employed in civilian positions outside of the CAF.

Although differences in concerns of personnel across DND/CAF components were minimal, other research has demonstrated that not all individuals, or groups of individuals, have been impacted in the same way or to the same extent by the pandemic. Although such differences were not explored in these analyses, other research in the DND/CAF has shown that women, younger personnel, and personnel with dependent children were most likely to be negatively affected by the COVID-19 pandemic (15, 74, 104). As such, it has been recommended that factors related to the unique challenges of Defence Team members continue to be applied in the development of organizational practices, policies, and programs (15). Moreover, given the suggestions for increased work-related flexibility made by respondents in this study, it is also suggested that supervisors and managers continue to monitor the needs and well-being of personnel and make allowances for employees' individual preferences and circumstances.

### Recommendations

In addition to identifying the main challenges they have faced, respondents were given the opportunity to make recommendations on how the DND/CAF might best support its personnel. One common recommendation was to improve and streamline communication regarding COVID-19, organizational responses to the pandemic, availability of support and services, and issues concerning career progression. To this end, Ivey et al. (105) recommended developing and implementing a consolidated and centralized location for communication that includes all relevant information in an accessible manner. Similarly, Sillins and Lee (106) emphasized the importance of communicating the threat of COVID-19 clearly in order to mitigate the risk of misinformation. Furthermore, Frank et al. (107) emphasized the importance of communicating information on the implications of the pandemic on career progression and job security. On the other hand, Mattke et al. (108) suggested that communication of support and services hosted by the organization should be made available through multiple and novel channels, rather than a single channel so that information may spread from multiple channels for maximum uptake. To ensure clarity and maximum update of communications, it is important that the organization ensure that communication be delivered through multiple accessible platforms and that the information delivered is consistent across platforms.

Another recommendation was related to the provision of technological support and training to better enable personnel to work from home. In addition to this, Ivey et al. (105) recommended to limit the number of new forms of technology to only those that are absolutely necessary, and ensure that these new systems are as user friendly as possible, particularly as employees adjust to teleworking.

Other recommendations made by respondents related to the need for managers to be understanding and express empathy for employees' needs, and to offer greater flexibility to accommodate those needs. This included recognizing a reduced work capacity and accommodating individual needs and circumstances. Similar recommendations were made in a variety of studies, including the suggestion that supervisors recognize parenting stressors and new roles at home and in the workplace (109) and inform personnel of childcare support services to help with individual circumstances (110, 111). Empathy on the behalf of supervisors and manager has been associated with reductions in employee somatic complaints and increased productivity (112), and has been found to mitigate the impact of difficult circumstances (e.g., wage cuts) on employee well-being (113).

One important consideration in this regard is to maximize to the degree possible employees' choices with respect to the return to their usual place of work. By allowing subordinates to decide to work in the location that best suits them, managers express an understanding and accommodation for individual situations, as well as confidence in their employees' abilities to work remotely. Moreover, the ability for employees to flexibly select their location for work, otherwise known as “work-from-anywhere” arrangements, can be advantageous to employee productivity (114).

Finally, while it was not specifically mentioned by personnel in the current study, research has highlighted the importance of the organization to promote physical fitness (115), for example, by encouraging personnel to use existing online support for indoor exercise programs (116). In the current study, less than half of participants reported engaging in physical exercise as a stress-management strategy. Encouraging physical exercise would aid in coping with the evolving situation of the COVID-19 pandemic and discourage sedentary behavior, which itself has been associated with anxiety, depression (81, 117, 118). A systematic review by Bentlage et al. (119) substantiated recommendations that individuals and their work teams organize comprehensive and feasible routine physical activity programs paired with digital technologies, virtual fitness programs, and relaxation protocols (i.e., indoor gardening, Tai Chi).

### Strengths and Limitations

The current study had a variety of strengths, including a very large sample size and inclusion of both close- and open-ended questions to further our understanding of the challenges and needs of employees during the COVID-19 pandemic. By asking respondents to describe their main challenges and thoughts on how the DND/CAF might address these challenges in their own words, we obtained individualized information on their needs and preferences, in addition to how they believe the organization can assist in this regard. The use of open-ended questions complemented results of quantitative analyses (15) and enabled participants to express their views in the context of their unique lived experiences.

Nevertheless, some study limitations must be acknowledged. First, because of the lack of probability-based sampling, the data is subject to self-selection bias to the degree that those who responded differed from non-respondents. In addition, because the open-ended questions were presented following close-ended survey questions, the content of close-ended questions may have drawn respondents' attention specifically toward similar, or parallel, elements of their personal experiences.

### Future Directions

As the situation with the COVID-19 pandemic is unprecedented and continues to evolve, the current findings raise novel directions for research on personnel well-being during the global pandemic. First, the survey was administered and collected in the early days of the pandemic, and many aspects of life and work may have changed since the start of the pandemic. Continued research is required to assess how challenges and concerns have changed since the summer of 2020. Moreover, senior members, including leaders and managers of the DND/CAF, are planning return-to-work arrangements for their subordinates. Therefore, research is required to determine and address the primary concerns military and civilian personnel have in regards to their return to their usual work locations.

Although only relatively minor differences were observed in the concerns of personnel from the three DND/CAF components, further research should investigate differences in individuals' lived experiences related to the COVID-19 pandemic along other characteristics (e.g., demographic characteristics, personality traits). Previous research has highlighted differences in concerns based on gender and family status (15). Gender differences in mental health have also been observed during the pandemic, such that women experienced greater levels of stress, anxiety and depression compared to men, and the degree of related functional impairment further varied according to individuals' family status (104).

## Conclusion

The current study reported aggregated responses from open-ended questions highlighting challenges to military and civilian defense personnel's work and well-being amid the COVID-19 pandemic. Responses to these open-ended questions highlighted important challenges to employees' productivity and overall well-being. Moreover, some of the challenges noted in the current study affected the well-being of regular force members, reservists, and civilians in public service to different extents, highlighting the importance of considering employees' roles and unique needs. Many of the reported challenges were not only similar to civilians in other sectors who have adapted to the COVID-19 pandemic, but were also consistent with other research on telework. Supporting personnel during this stressful time is essential to employee well-being, productivity, and overall organizational effectiveness.

## Data Availability Statement

The dataset presented in this article are not readily available because the data was collected solely for the purposes and may not be shared externally or used for other purposes as per the informed consent used for this study. In addition, the data are property of her Majesty The Queen, Department of National Defense and the Government of Canada and Department of National Defense Canada: http://forces.gc.ca/.

## Ethics Statement

The studies involving human participants were reviewed and approved by the Social Science Research Review Board, managed by the Director General Military Personnel Research and Analysis in the Department of National Defense. The patients/participants provided informed consent to participate in this study.

## Author Contributions

IG contributed to the conception and administration of the COVID-19 Defence Team Survey, the analysis of qualitative responses, and the writing and editing of the manuscript. WD contributed to the writing and editing of the manuscript and the creation and formatting of the tables. JL contributed to the conception administration of the COVID-19 Defence Team Survey, the analysis of qualitative responses, and editing of the manuscript. All authors contributed to the article and approved the submitted version.

## Conflict of Interest

The authors declare that the research was conducted in the absence of any commercial or financial relationships that could be construed as a potential conflict of interest.

## Publisher's Note

All claims expressed in this article are solely those of the authors and do not necessarily represent those of their affiliated organizations, or those of the publisher, the editors and the reviewers. Any product that may be evaluated in this article, or claim that may be made by its manufacturer, is not guaranteed or endorsed by the publisher.
