# Supplementary material for: Pandemic-Related Challenges and Organizational Support Among Personnel in Canada's Defense Establishment
Source: Front Public Health. 2022 Jan 27;9:789912. doi: 10.3389/fpubh.2021.789912 (PMC8829139; doi:10.3389/fpubh.2021.789912)
Supplement: Supplementary file 1 [file Table_1.DOCX]

**Supplementary materials**

Table S1

*Frequency and percentage of respondents who mentioned each work-related challenge*

| Theme | Frequency | Percentage of respondents |
| --- | --- | --- |
| 1. Dissatisfaction with technology/software | 5,038 | 27.1 |
| 1. Dissatisfaction with working arrangement | 3,810 | 20.5 |
| 1. Ergonomic or work equipment/resources | 2,659 | 14.3 |
| 1. Work and life/family balance | 2,439 | 13.1 |
| 1. Communication challenges | 2,155 | 11.6 |
| 1. Increase in work volume | 1,177 | 6.3 |
| 1. Effects on career development | 1,144 | 6.1 |
| 1. Lack of PPE/testing equipment/safety protocols | 854 | 4.6 |
| 1. DND/CAF information/messaging | 757 | 4.1 |
| 1. Leadership and CoC | 724 | 3.9 |
| 1. Uneven distribution of work among personnel | 700 | 3.8 |
| 1. Decrease in work volume | 665 | 3.6 |
| 1. Support and management of employees remotely | 659 | 3.5 |
| 1. Teamwork difficulties | 657 | 3.5 |
| 1. Job/financial security | 626 | 3.4 |
| 1. Return to work concerns | 543 | 2.9 |
| 1. Social ties/isolation | 497 | 2.7 |
| 1. Limited opportunities to exercise | 382 | 2.1 |
| 1. Changes in work responsibilities | 372 | 2.0 |
| 1. Posting uncertainty | 348 | 1.9 |
| 1. Fear of contracting COVID -19 | 311 | 1.7 |
| 1. Supervisor /immediate manager | 287 | 1.5 |
| 1. Concerns with DND/CAF’s response to COVID-19 | 267 | 1.4 |
| 1. Spouse/partner’s job/financial security | 164 | 0.9 |
| 1. Issues working with contractors and/or clients | 119 | 0.6 |
| 1. Effects on leave | 84 | 0.5 |
| 1. Unavailability of routine health services | 66 | 0.4 |
| 1. 699 leave concerns | 19 | 0.1 |
| 1. Other | 1,487 | 8.0 |
| 1. N/A | 522 | — |

Table S2

*Frequency and percentage of respondents who mentioned each personal and family challenge*

| Theme | Frequency | Percentage of respondents |
| --- | --- | --- |
| 1. Social isolation | 7,089 | 38.7 |
| 1. Mental health | 2,438 | 13.3 |
| 1. School closures & homeschooling | 2,208 | 12.1 |
| 1. Parents/elderly family members | 1,904 | 10.4 |
| 1. Concern over loved ones contracting COVID-19 | 1,848 | 10.1 |
| 1. Childcare concerns | 1,792 | 9.8 |
| 1. Work-life balance | 1,590 | 8.7 |
| 1. Family stress due to confinement | 1,562 | 8.5 |
| 1. Children’s well-being | 1,422 | 7.8 |
| 1. Balancing work and childcare | 1,206 | 6.6 |
| 1. Finances | 1,148 | 6.3 |
| 1. Limited opportunities for exercise | 1,105 | 6.0 |
| 1. Marital/relationship conflict | 938 | 5.1 |
| 1. Physical health | 746 | 4.1 |
| 1. Spouse/partner’s workload | 708 | 3.9 |
| 1. Spouse/partner’s employment | 628 | 3.4 |
| 1. Grocery shopping | 612 | 3.3 |
| 1. Cancelling/postponing a significant event | 602 | 3.3 |
| 1. Fear of personally contracting COVID-19 | 601 | 3.3 |
| 1. Unavailability of routine health services | 590 | 3.2 |
| 1. Adult children well-being | 582 | 3.2 |
| 1. General satisfaction | 480 | 2.6 |
| 1. Managing postings | 387 | 2.1 |
| 1. Increase in work volume | 381 | 2.1 |
| 1. Selling/buying a home | 299 | 1.6 |
| 1. Managing shared custody of children | 169 | 0.9 |
| 1. Effects on leave | 166 | 0.9 |
| 1. Ergonomics of work setup at home | 135 | 0.7 |
| 1. Reduced personal freedoms | 110 | 0.6 |
| 1. Ability of health care system to address needs | 64 | 0.3 |
| 1. Other | 1,141 | 6.2 |
| 1. N/A | 238 | — |

Table S3

*Frequency and percentage of respondents who mentioned each stress management strategy*

| Theme | Frequency | Percentage of respondents |
| --- | --- | --- |
| 1. Exercise | 7,215 | 40.9 |
| 1. Time outdoors | 4,750 | 26.9 |
| 1. Spending time with immediate family or pet | 2,084 | 11.8 |
| 1. Communicating with friends/family/co-workers | 1,865 | 10.6 |
| 1. Household chores/house projects | 1,843 | 10.4 |
| 1. Mind-body wellness/relaxation | 1,723 | 9.8 |
| 1. Playing games | 1,340 | 7.6 |
| 1. Creating a routine/setting goals | 1,058 | 6.0 |
| 1. Reading or listening to audiobooks/podcasts | 1,024 | 5.8 |
| 1. Social support from friends or family | 930 | 5.3 |
| 1. Cooking/baking or eating well | 913 | 5.2 |
| 1. Watching movies, television, online media | 812 | 4.6 |
| 1. Music, singing, or playing instruments | 747 | 4.2 |
| 1. Positive mentality | 666 | 3.8 |
| 1. Engaging in interests/hobbies/passions | 611 | 3.5 |
| 1. Artistic activities | 577 | 3.3 |
| 1. Sleep | 546 | 3.1 |
| 1. Alone/quiet time | 495 | 2.8 |
| 1. Mastery/learning activities | 372 | 2.1 |
| 1. Avoiding the news | 363 | 2.1 |
| 1. Taking breaks or limiting work hours | 354 | 2.0 |
| 1. Working | 350 | 2.0 |
| 1. Practicing religion/spirituality | 341 | 1.9 |
| 1. Going for a drive | 272 | 1.5 |
| 1. Drinking alcohol | 259 | 1.5 |
| 1. Writing (e.g., in journal) | 186 | 1.1 |
| 1. Cannabis use | 163 | 0.9 |
| 1. Following COVID-19 guidelines/protocols | 155 | 0.9 |
| 1. Keeping up to date with COVID-19 information | 153 | 0.9 |
| 1. Detaching from electronic devices | 152 | 0.9 |
| 1. Professional help/counselling | 134 | 0.8 |
| 1. Social support from co-workers or supervisor | 131 | 0.7 |
| 1. Hygiene (bathing and showering) | 94 | 0.5 |
| 1. Volunteering | 83 | 0.5 |
| 1. Shopping (online or in-person) | 65 | 0.4 |
| 1. Using other drugs | 56 | 0.3 |
| 1. Smoking cigarettes and/or cigars | 44 | 0.2 |
| 1. Other | 2,004 | 11.4 |
| 1. N/A | 174 | — |

Table S4

*Frequency and percentage of respondents who mentioned each theme relating to how the DND/CAF can better support work during the COVID-19 pandemic*

| Theme | Frequency | Percentage of respondents |
| --- | --- | --- |
| 1. Improve DWAN or DVPNI | 2,582 | 26.6 |
| 1. Clarify /streamline communications | 1,527 | 15.7 |
| 1. General satisfaction | 1,372 | 14.1 |
| 1. Provide hardware for remote work | 886 | 9.1 |
| 1. Recognize reduced work capacity | 519 | 5.3 |
| 1. Support virtual teamwork structures | 469 | 4.8 |
| 1. Flexibility for work location/hours | 447 | 4.6 |
| 1. Communicate return-to-work timelines and procedures | 415 | 4.3 |
| 1. Support at-home workspaces | 356 | 3.7 |
| 1. Improve financial compensation | 338 | 3.5 |
| 1. Distribute work fairly | 314 | 3.2 |
| 1. Recognize domestic responsibilities | 281 | 2.9 |
| 1. Provide PPE | 242 | 2.5 |
| 1. Improve IT support services | 236 | 2.4 |
| 1. Integrate various remote work software | 223 | 2.3 |
| 1. Establish sanitary precautions for return to work | 214 | 2.2 |
| 1. Better define essential and non-essential work | 159 | 1.6 |
| 1. Improve work-life balance | 135 | 1.4 |
| 1. Support childcare access | 132 | 1.4 |
| 1. Improve intradepartmental synergy | 130 | 1.3 |
| 1. Establish social distancing procedures | 115 | 1.2 |
| 1. Support mental health | 108 | 1.1 |
| 1. Support physical/medical health | 108 | 1.1 |
| 1. Support physical fitness | 93 | 1.0 |
| 1. Improve security for remote work | 81 | 0.8 |
| 1. Provide access to essential hardware at workplace | 40 | 0.4 |
| 1. Other | 1,063 | 10.9 |
| 1. N/A | 274 | — |

Table S5

*Frequency and percentage of respondents who mentioned each theme relating to how the DND/CAF can better support personal and family needs during the COVID-19 pandemic.*

| Theme | Frequency | Percentage of respondents |
| --- | --- | --- |
| 1. General satisfaction | 1,597 | 23.5 |
| 1. Improve communication in general | 760 | 11.2 |
| 1. Support flexible work arrangements | 536 | 7.9 |
| 1. Support telework/remote work arrangements | 426 | 6.3 |
| 1. Expand benefits/entitlements | 397 | 5.8 |
| 1. Support childcare access | 394 | 5.8 |
| 1. Consideration for childcare and homeschooling | 371 | 5.5 |
| 1. Communicate empathy, understanding, and support | 362 | 5.3 |
| 1. Change travel restrictions | 307 | 4.5 |
| 1. Improve work-life balance | 278 | 4.1 |
| 1. Improve current leave policy | 274 | 4.0 |
| 1. Improve management of postings | 265 | 3.9 |
| 1. Recognize reduced work capacity | 263 | 3.9 |
| 1. Establish health and safety precautions for return to work | 249 | 3.7 |
| 1. Improve financial compensation | 221 | 3.3 |
| 1. Support mental health | 171 | 2.5 |
| 1. Strengthen IT infrastructure | 137 | 2.0 |
| 1. Support physical/medical health | 125 | 1.8 |
| 1. Communicate available benefits/entitlements | 125 | 1.8 |
| 1. Provide job security information/reassurance | 115 | 1.7 |
| 1. Support physical fitness | 108 | 1.6 |
| 1. Distribute workload fairly | 85 | 1.3 |
| 1. Provide PPE | 81 | 1.2 |
| 1. Provide accommodations for medically vulnerable | 72 | 1.1 |
| 1. Provide information about leave code 699 | 65 | 1.0 |
| 1. Provide information about leave policy | 26 | 0.4 |
| 1. Other | 457 | 6.7 |
| 1. N/A | 310 | — |
